# In silico method for systematic analysis of feature importance in microRNA-mRNA interactions

**DOI:** 10.1186/1471-2105-10-427

**Published:** 2009-12-16

**Authors:** Jiamin Xiao, Yizhou Li, Kelong Wang, Zhining Wen, Menglong Li, Lifang Zhang, Xuanmin Guang

**Affiliations:** 1 College of Chemistry, Sichuan University, Chengdu 610064, PR China; 2 Department of Chemistry and Biochemistry, Texas Tech University, Lubbock, Texas 79409-1061, USA

## Abstract

**Background:**

MicroRNA (miRNA), which is short non-coding RNA, plays a pivotal role in the regulation of many biological processes and affects the stability and/or translation of mRNA. Recently, machine learning algorithms were developed to predict potential miRNA targets. Most of these methods are robust but are not sensitive to redundant or irrelevant features. Despite their good performance, the relative importance of each feature is still unclear. With increasing experimental data becoming available, research interest has shifted from higher prediction performance to uncovering the mechanism of microRNA-mRNA interactions.

**Results:**

Systematic analysis of sequence, structural and positional features was carried out for two different data sets. The dominant functional features were distinguished from uninformative features in single and hybrid feature sets. Models were developed using only statistically significant sequence, structural and positional features, resulting in area under the receiver operating curves (AUC) values of 0.919, 0.927 and 0.969 for one data set and of 0.926, 0.874 and 0.954 for another data set, respectively. Hybrid models were developed by combining various features and achieved AUC of 0.978 and 0.970 for two different data sets. Functional miRNA information is well reflected in these features, which are expected to be valuable in understanding the mechanism of microRNA-mRNA interactions and in designing experiments.

**Conclusions:**

Differing from previous approaches, this study focused on systematic analysis of all types of features. Statistically significant features were identified and used to construct models that yield similar accuracy to previous studies in a shorter computation time.

## Background

MicroRNAs (miRNAs) are short non-coding RNAs of approximately 22 nucleotides with some differences in one or two nucleotides in the 3' terminus. In eukaryotes, miRNA affects the stability and/or translation of mRNAs and is involved in the regulation of various biological processes, such as development, differentiation, and apoptosis [1-5]. It has been reported that more than one-third of human genes can be targeted by miRNA and miRNAs have been linked to conditions such as lymphoma, leukemia, and lung adenocarcinoma [6,7]. Stage-specific, tissue-specific and relatively low expression results in considerable miRNA complexity. Thus, identification of the functions of miRNA is an important and challenging problem.

Although bioprocesses involving miRNA-mRNA interactions, such as cleavage and translational repression of target mRNA, depending on the degree of base pairing between the miRNA and the mRNA sequence, are understood, actual correlation and the mechanism of these interactions are still unclear. Since miRNA *lin-4 *and *let-7 *were discovered in *Caenorhabditis elegans*, there has been a huge focus on this field and a large number of miRNAs have been identified in various species [8-11]. There are 6211 mature miRNA sequences in the current miRBase sequence database (release 11.0) [12]. Despite this large number of miRNAs identified, only a few miRNA targets are known. According to TarBase 4.0, there are only 763 experimentally validated target sites, which is much less than the number of miRNA sequences [13], so target identification is important in understanding the mechanism and biological functions of miRNA-mRNA interactions.

Since the first miRNA target prediction algorithm was published [14], an increasing number of computational algorithms have been developed for this purpose. Three main types of features have been successfully applied in these algorithms: the complementarity of the seed region in the 5' terminus, thermodynamic stability, and cross-species conservation [15-18]. However, researchers had to designate a few arbitrary kilobases downstream from the stop codon when an experimentally validated 3' untranslated region (UTR) was lacking for certain species [19]. The thermodynamic stability is useful for secondary structure prediction since miRNA binds to the RNA-induced silencing complex to form a large protein complex. Moreover, experiments have revealed that approximately 30% of miRNAs do not exhibit cross-species conservation [20,21].

Hence, machine learning algorithms were developed and shed light on the prediction of miRNA targets. Based on sequence information, TargetBoost refined some significant features to improve the performance of model and was capable of predicting more actual target genes [22]. By extracting similar features from experimental data, miTarget and NBmiRTar were developed using a support vector machine and a naïve bayes approach, respectively [23,24]; both yielded satisfactory prediction results when artificial negative data were used for model training.

An ensemble prediction algorithm for human miRNA targets developed using absolute experimentally validated data yielded a cross-validation (CV) accuracy of 82.95% [25]. However, through rigorous selection, only 48 positive and 16 negative samples were used for training. Another algorithm, MiRTif, was released with 195 positive and 38 negative experimentally validated target sites, for which a duplex binding picture for prediction by RNAhybrid was available for 17 new negative samples. The algorithm achieved sensitivity of 83.59% and specificity of 73.68% [26]. However, the current set of experimentally validated negative samples is insufficient to represent the negative class and therefore more negative data are required. Hence, two negative data sets were generated in our study.

Microarrays can also provide many experimental data for training models. Recently, several studies reported on miRNA target prediction from microarray data analysis [27,28]. MirTarget2, which was developed based on microarray data, is considered to have great potential for high-throughout target validation by transcriptional profiling and improved miRNA target prediction, with a result of 0.79 for the area under the receiver operating characteristic curve (AUC) [29].

**Figure 1:**
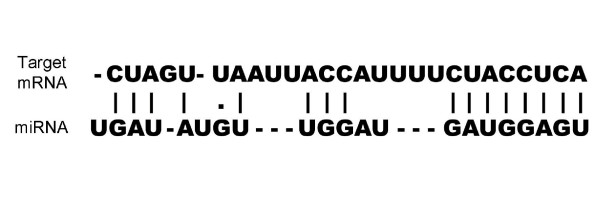
**Simplified picture of miRNA-target interactions**.

In the present study, systematic analysis of feature importance was performed based on permutation importance and conditional variable importance strategies. A random forest (RF) approach was applied for prediction of miRNA-target interactions. Three types of features were considered, sequence, structural, and positional features. These features were extracted from binding pictures of miRNA-target duplexes and regarded as a unit (Figure 1) instead of being artificially divided into two segments. This approach might well preserve the actual biological properties. For each single feature set and the whole hybrid feature set, model training was repeated 100 times. The models yielded high sensitivity and specificity and the feature importance scores were then calculated. Only statistically significant features were used to refine the models, which yielded similar accuracy to that obtained in previous studies. Our results indicate that these features significantly contribute to the performance of the model and will help in reducing the number of experimental procedures required in research into miRNA-target interactions.

## Results and Discussion

### Feature extraction

All features were classified as sequence, structural or positional features. Studies have demonstrated that thermodynamic stability plays an important role in miRNA target prediction and machine learning algorithms have confirmed this [30,31]. In our feature extraction procedure, several features relate to thermodynamic feature indirectly, because the RNA secondary structure prediction was based on thermodynamic properties. Target sites for seed and non-seed segments were treated artificially in previous studies, which destroy the integrity of the target site and leads to underinvestigation of relevant biological properties. Here, a miRNA-target duplex was treated as a unit, which overcomes the disadvantage of previous methods, and feature extraction was parsed using *Perl*.

Sequence features (SEQ, Table 1) mainly include base frequencies and compositions. Background frequencies (*p*_A _= 0.34, *p*_C _= 0.19, *p*_G _= 0.18, *p*_U _= 0.29) have typically been used to produce artificial negative samples [22,32]. In microarray data analysis, researchers discovered that the four base frequencies were significantly different for candidate downregulated and normal genes. Furthermore, many dinucleotide sequences are statistically significant in miRNA target prediction [27,29].

**Table 1:** Sequence features

Feature	Description
N_frac	Four features, percentage of A U C G nucleotides in the target sequence
GC_frac	Percentage of GC content in the target sequence
nt_match	Percentage of matching bases in the target sequence
nt_GUmatch	Percentage of GU matching bases in the target sequence
nt_mismatch	Percentage of mismatch bases in the target sequence
Dinucleotides	16 features, number of dinucleotide counts in the target sequence
Trinucleotides	64 features, number of trinucleotide counts in the target sequence

Structural features (STRU, Table 2), including folding information for miRNA-target duplexes, should have the necessary information and should be crucial for recognition of miRNA-target interactions. Three main types of structural features, stems, loops and bulges, were used to represent RNA secondary structure. A well-known perfect or near-perfect pairing seed region is fairly important for these interactions (G:U wobble base-pairing is allowed). In addition, various types of loops and bulges in both seed and non-seed regions also affect the interactions [33].

**Table 2:** Structural features

Feature	Description
Stem	Number of stems
Overhang	Number of overhangs
Stem_max	Length of the maximal stem in the target sequence
Loop_mismatch	Number of loops that only contain two mismatch bases
Loop_symm	Number of symmetric loops that contain more than two bases
Loop_asym	Number of asymmetric loops that contain more than two bases
Stems^a^	8 features, number of stems of length 6-0 and those with length < 6 and > 10
Loops^b^	8 features, the number of loops with length of 1-7 and those with length > 7
Bulges^c^	8 features, number of bulges with length of 1-7 and those with length > 7

^a ^A stem is defined as a set of consecutive pairs separated by unpaired bases.

^b ^A loop is defined as a set of unpaired bases between two strands.

^c ^A bulge is defined as a set of unpaired bases only in one strand

Positional features (POSI, Table 3) reflect the mechanism of miRNA-target interactions. Saunders *et al*. investigated polymorphism of miRNA-target duplexes using single nucleotide polymorphism data, which revealed that a base mutation in the target sequence affects the regulation function of miRNA [34]. These studies suggest that position-specific states (whether matching or not) may be important for miRNA target identification. Here only 21 nucleotides from the 5' terminus were considered. Four binary numbers were used to represent a position. If the sequence length is < 21 nt, the RF algorithm can automatically set the missing positions as missing values and replace them with the most frequent non-missing value (see the Methods section).

**Table 3:** Positional features

First two binary numbers	Meaning	Last two binary numbers	Meaning
00	A	00	Mismatch
11	U	11	GU match
10	G	10	Match
01	C		

### Prediction performance with RF

We used the RF algorithm and optimized the parameters. Two parameters, number of trees to grow *ntree *and number of variables randomly sampled as candidates at each split *mtry *were optimized using a grid search approach. During the grid search, the values of *ntree *= {500, 2000, 500} and *mtry *= {0, mdim, 1} were optimized based on 10-fold cross-validation (where the first number indicates the initial value, the second indicates is the final value, and the third is the increment used to generate values; mdim is number of features), which is partitions the original sample into 10 subsamples, 9 subsamples are to train model and the remainder one to test model and this process repeats 10 times. We then selected the value for the best-performing parameters to estimate the performance of the training model. It has been reported that an out-of-bag (OOB) error is very similar to the classification error for cross-validation (see Methods), which is a built-in measure of performance [35]. Table S1 lists the predictor performance comparison between based on cross-validation and OOB estimate (see Additional file 1).

RF models were constructed using SEQ, STRU, POSI, and the total feature set for two difference negative data sets; 200 samples from each class were randomly selected and used for training. This procedure was repeated 100 times and the average fraction of true positive (sensitivity) and true negative (specificity) predictions were used to determine the accuracy (The 200 negative samples always contain 35 experimental data in every randomly selection). The average prediction results for the models are listed in Table 4. Models based on the negative data set Neg_1 yielded higher accuracy than those based on Neg_2, except for specificity for SEQ and POSI, probably because of the different data sources. The performance using sequence or structural features was worse than that for positional features. Our models achieved sensitivity of 0.947 and specificity of 0.916 for Neg_1 and sensitivity of 0.917 and specificity of 0.949 for Neg_2 using positional features. These results indicate that positional features yield a low number of false positive predictions and good model performance. Several studies have clearly shown an increase in the accuracy of prediction on combining numerous features. However, the hybrid of different feature types did not yield the highest accuracy (sensitivity 0.870 and specificity 0.922) for Neg_2. This indicates that negative correlation occurs in hybrids of different features. Hence, we examined the interactions among different features in each set using correlation analysis (see Additional file 2).

**Table 4:** RF prediction results

Feature set	Neg_1			Neg_2		
	
	Optimal parameters	Se	Sp	Optimal parameters	Se	Sp
SEQ	ntree^a ^= 2000, mtry^b ^= 16	0.873	0.828	ntree = 1000, mtry = 66	0.821	0.885
STUR	ntree = 1500, mtry = 16	0.852	0.826	ntree = 1500, mtry = 7	0.807	0.808
POSI	ntree = 1000, mtry = 5	0.947	0.916	ntree = 1000, mtry = 4	0.917	0.949
Total	ntree = 2000, mtry = 6	0.971	0.918	ntree = 500, mtry = 37	0.870	0.922

Cross-validation was used to estimate the predictor performance of SEQ, STRU, POSI sets and the total feature set for two differet negative data sets. Neg_1 comprises all experimental samples and inferred negative samples and Neg_2 comprises all experimental samples and artificial negative samples from miRanda. Sensitivity (Se) was calculated as TP/(TP+FN) and specificity (Sp) as TN/(TN+FP), where TP is the number of true positives, TN is the number of true negatives, FP is the number of false positives and FN is the number of false negatives.

^a ^number of trees to grow.

^b ^number of variables randomly sampled as candidates at each split.

#### Feature importance measures

Current classification tasks need a measure of feature importance rather than only predicting the response using "black-box" models. Here, two different strategies were applied to measure feature importance in the prediction of miRNA-target interactions.

#### Permutation importance analysis of RF

**Figure 2:**
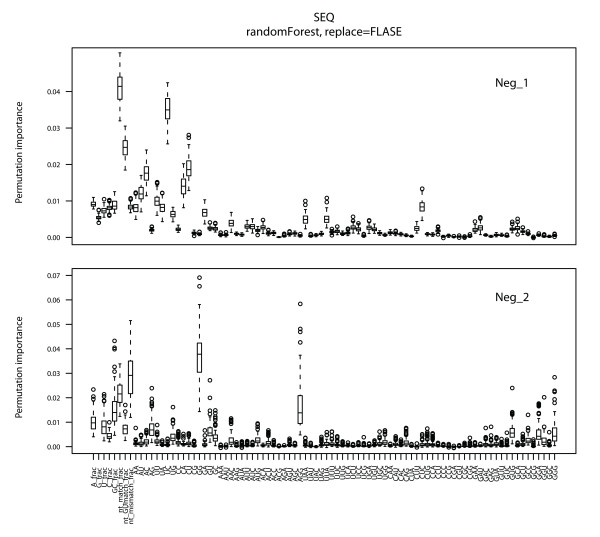
**Feature importance measure for sequence features**. The discriminatory power of each feature was determined by calculating the importance value, with larger values indicating more relevant properties. The importance distribution is shown for each sequence feature as a box plot in which the middle bar is the median, the outer edges are the 10 and 90 percentiles and the edges of the box are the 25 and 75 percentiles. Outliers are shown as circles. Neg_1 (all experimental samples and inferred negative samples) and Neg_2 (all experimental samples and artificial negative samples from miRanda) were analyzed separately.

**Figure 3:**
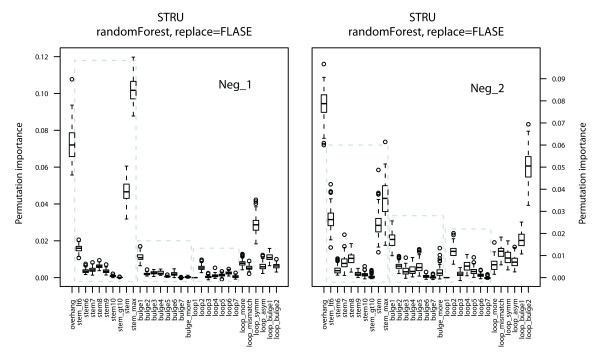
**Feature importance measure for structural features**. The three rectangles denote features related to stems, bulges and loops.

**Figure 4:**
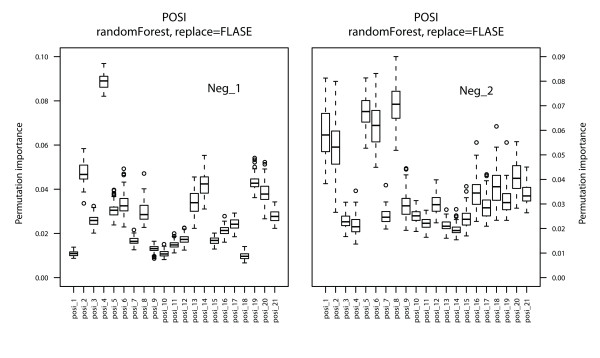
**Feature importance measure for positional features**.

RF is a classification method that also provides feature importance measures. It can distinguish significant predictor features from uninformative features and reduces interactions among features as much as possible. Three measures of feature importance, the selection frequency, Gini importance and permutation importance, are available. In the present study, permutation importance was used as to measure feature importance in miRNA-target interactions separately for three feature sets to distinguish significant functional predictor features. The process was repeated 100 times with random resampling of constructed models and the feature measure scores were calculated. The distributions of these scores are shown in Figures 2, 3 and 4.

For sequence features (Figure 2), the percentage of match bases greatly contributes to recognition of interactions for both Neg_1 and Neg_2. The results are consistent with those of previous studies [27,29,36]. In addition, several dinucleotide and trinucleotide sequences were also statistically significant, such as UC, GG, CUC, and AGG. However, probably because of the negative data sources, base frequencies for A, G, and U in Neg_2 were ranked in the top 10. The results indicate that sequence-based feature importance depends on the data source.

The most informative structural feature was the stem, which indicated matching conditions for both seed and non-seed regions. Bulges provide more insight into miRNA-target interactions than loops do because more informative features are relate to bulges than to loops (Figure 3). The overhang also has a significant effect on classification, which depends on the nature of the miRNA-target duplex. Features related to stems revealed that a length of 8 nt may be a more suitable definition of the seed region, although false miRNA targets usually had a match of < 6 nt in the seed region. Moreover, 1-nt bulges and 2-nt loops were ranked top and thus might greatly affect miRNA-target interactions.

Positional features suggested that non-seed regions also play an important role in miRNA-target interactions as shown by Figure 4, in which matching/mismatching serve as the positive/negative class, respectively. It has been shown in many studies that perfect or near-perfect base pairing in the seed region greatly contributes to the performance of models. Our results confirm that most of the dominant functional positional features are in the seed region.

#### A new feature importance measure strategy using hybrid models

A further investigation was implemented for a combination of the three feature sets. In this section, a novel conditional feature importance strategy was used to evaluate the total features. This strategy was implemented using the *cforest *function of the *party *package in R language, which can generate additional information on feature interactions [37-39].

**Figure 5:**
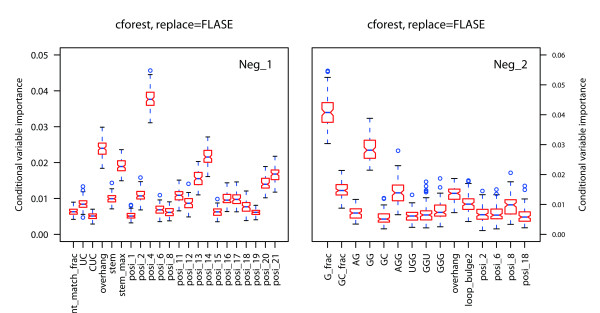
**Comprehensive evaluation results**. The plot displays the distribution for conditional feature importance using the *cforest *function for median importance values > 0.005. Sample without replacement and return unscaled measurement.

A combination of three feature sets was considered to evaluate the feature importance using conditional variable importance strategy. Training data were randomly sampled and the procedure was repeated 100 times as above. Some of the significant features are shown in Figure 5. The measure strongly indicates that positional features greatly contribute to miRNA-target interactions because most statistically significant features are positional features in Neg_1. However, sequence features are dominant in the statistically significant features in Neg_2. These results are in agreement with the RF permutation importance. In addition, this strategy based on conditional inference trees seems to produce less noise than a permutation importance strategy.

#### Prediction performance of using only statistically significant features

To demonstrate the statistically significant features for predicting miRNA-target interactions, we compared the performance of different feature sets using only the significant features for two different negative data sets. The procedure is summarized below.

(1) Calculate the feature importance score for each feature and rank the features according to these scores.

(2) Eliminate the last feature and use the remaining features to construct a model.

(3) Repeat step 2 until a remarkable decrease in accuracy occurs.

(4) The remaining features are considered statistically significant and are used to construct a model. Receiver operating characteristic (ROC) are used to evaluate the prediction sensitivity and specificity.

**Figure 6:**
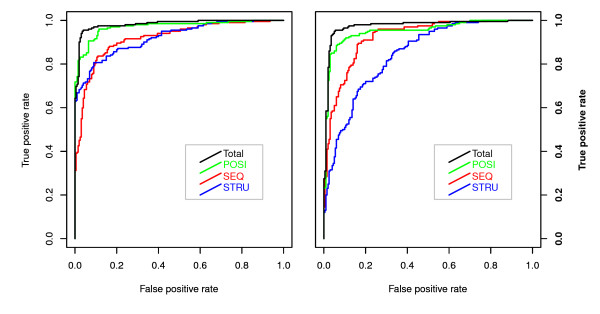
**ROC curves for miRNA-target interactions identified using Neg_1 and Neg_2**. Models were developed using only 10, 11, 5 and 10 statistically significant sequences, structural and positional features and total features, respectively. ROC curves are used to evaluate and compare the performance of miRNA-target interactions identified for four different feature sets.

We used this procedure to analyze the contribution of statistically significant features to the prediction accuracy (see Additional file 3). Finally, the top 10, 11, 5, and 10 statistically significant features were used to construct models for SEQ, STRU, POSI and total feature sets, which yielded AUC values of 0.919, 0.922, 0.969, and 0.978 for Neg_1 and 0.926, 0.874, 0.954, and 0.970 for Neg_2, respectively. Figure 6 shows ROC curves for each feature set for the two data sets. The highest accuracy was obtained by combining three types of feature sets. It is clear that the positional feature set exhibits higher accuracy than the other two feature types. The positional features were first investigated using miTarget, in which there are five position-based features ranked in the top 10, all belonging to the seed region. However, our positional features include two pieces of information: the type of nucleotide and whether it matches or not.

In NBmiRTar, most statistically significant features were associated with loops and bulges, similar to the structural features of our method. Its motif features correspond to dinucleotide and trinucleotide sequences in our method. Our results prove that stems greatly contribute to recognition of miRNA-target interactions. More systematic analysis of dinucleotide and trinucleotide sequences was carried out in this study. MiRTif uses various *k*-gram frequencies as features for a triplet SVM classifier to predict pre-miRNA [40,41]. It is thought that these features represent the real environment for miRNA-target interactions. However, they might not be suitable for guiding experimental procedures.

## Conclusions

MiRNA investigation not only sheds new light on RNA function, but can also reveal the mechanism involved in cell function and regulation. The actual correlation and mechanism for miRNA-target interactions are still unclear. However, the best solution might involve a combination of experimental and computational approaches. Our results demonstrate that this method yields good prediction and is robust. Moreover, the results will be useful in designing experimental procedures. As more experimental and unbiased data become available, our approach could be improved and used to identify more reliable predictor features reflecting real miRNA-target interactions.

## Methods

Machine learning, a broad subfield of artificial intelligence, can be used to automatically extract general rules from data sets through experience. Random forest is one of the most accurate prediction tools currently available for classification and regression. It is briefly described in this section.

### Random forest

Random forest (RF) contains a number of unpruned decision trees. Each tree is trained and gives a classification using a different bootstrap sample from the original data. RF does not need a separate test set to obtain an unbiased estimate of the test set error because when using bootstrap sample from the original data, approximately one-third of the samples are left for internal estimation, which is called OOB data. However, if measures are based on the predictors' performance in the training set, there is no way of knowing whether the predictors are over-fitted to the training set. Instead, cross-validation should be used to test the performance of predictors. The RF algorithm is widely used for classification and regression. It has been applied in complicated interactions and for data sets with many features, or so-called "small *n*, large *p*" problems [35,42]. Based on a tree structure, it has some advantages, such as interpretable classification rules and additional information that measures the importance of features. The important feature extraction strategy is a difficult issue owing to the complexity of feature interaction with other features. However, prediction of the model response cannot be achieved for many applications. Furthermore, RF can replace missing values by computing the median of all values of a variable in class *j *when the *m*th variable is not categorical, then using this value to replace all missing values for the *m*th variable in class *j*. If the *m*th variable is categorical, replacement is for the most frequent non-missing value in class *j*. These replacement values are called fills [35].

The rationale for permutation importance is random permutation of the predictor variable *X*_*j*_, so its original association with the response *Y *disappeared. When the permutated variable *X*_*j*_, together with the remaining non-permutated predictor variables, is used to predict the response, the prediction accuracy (i.e. the number of observations classified correctly) decreases greatly if the original variable *X*_*j *_is associated with the response. Thus, a reasonable measure for variable importance is the difference in prediction accuracy before and after permutation of *X*_*j*_. As an improvement, conditional importance can be considered [37,39]. Feature importance was our main focus, which is suitable for feature selection in many applications. In this study we used the *randomForest *and *party *packages in R language.

### Data sets and performance evaluation

All experimental data were downloaded from TarBase 4.0 [11], which records experimentally validated target data via manual collection. The criteria for selection of training data were as follows:

(1) Cleaved target data were eliminated because they might be different from translation repressed targets.

(2) A miRNA-target duplex binding picture must be available (Figure 1).

(3) Each target site sequence should not contain any unknown nucleotide (i.e. N can represent any nucleotide).

Consequently, a total of 294 miRNA-target pairs (259 positive and 35 negative) were collected for six species: Drosophila, Caenorhabditis elegans, human, mouse, rat and zebrafish. These data contained folding information for duplexes, and truly biologically relevant simulation adapts to feature importance measures. However, the current set of 35 validated negative samples might not be enough to represent the negative class (Table S2 in Additional file 4). Therefore, two artificial negative sets were generated, as described below.

An inferred negative sample set and all negative experimental samples comprised Neg_1. It has been reported that *let-7 *miRNA cannot repress expression after deletion of target sites on *lin-41 *[23]. In other words, the remaining regions on the *lin-41 *3' UTR are not targeted by *let-7 *miRNA [33]. Thus, if all the actual target sites on *lin-41 *are masked, then the other remaining regions with favorable seed pairings are apposite as negative samples. The same is true for miR-126* [43]. In practice, we used RNAhybrid to predict the binding duplex, using only duplexes with a match > 4 nt and discarding the other pairs to improve the quality of the data set. Thus, 167 inferred negative samples were obtained, 65 from *let-7 *and 102 from miR-126*.

We also generated 1000 artificial mature miRNAs (20-24 nt long) with A, C, G and U frequencies of 0.34, 0.19, 0.18 and 0.29, respectively. These base frequencies are not consistent with those in true miRNA. MiRanda was then used to predict target sites for the 1000 artificial miRNA from the 12,102 human 3'UTR sequences [44]. All these target sites are presumed to be false positive predictions since the query sequence did not include true miRNA. In practice, only 50 random artificial miRNA sequences were used to generate artificial negative samples from the 1000 sequences, because use of all 1000 artificial sequences yielded a set of predictions that was too large to be manageable. This artificial negative set was produced using the default MFE and SC value. In this case, MiRanda produced 78,169 false target sites, which together with the experimental negative samples comprised NEG_2. TargetBoost and NBmiRTar also generated a large negative class with this method [22,24].

Finally, the sensitivity and specificity were evaluated. The sensitivity for positive prediction, specificity for negative prediction and ROC plots of the true positive rate versus the false positive rate for varying decision cutoffs were used as measures of the model performance.

## Availability and requirements

Source code and binaries freely available for download at http://cic.scu.edu.cn/bioinformatics/miRNA_code.zip

Programming language: Perl, R language

License: none

## Abbreviations

miRNA: microRNA, mRNA, messenger RNA; RF: Random forest; SEQ: sequence features; STRU: structural features; POSI: positional features.

## Authors' contributions

JX wrote the programs, designed the experiments, and drafted the manuscript. YL, LZ, XG helped in analysis and discussion, YL, KW, ZW refined the manuscript and gave useful comments. ML initialized and supervised the whole project. All authors read and approved the final manuscript.

## Supplementary Material

Additional file 1 **Table S1**. Comparison of OOB and CV error estimate. Click here for file

Additional file 2 The interactions among different features in each set using correlation analysis. Click here for file

Additional file 3 The analysis on contribution of statistically significant features. Click here for file

Additional file 4 **Table S2**. Prediction results of RF using experimental data. A very imbalanced experimental dataset (259 positive and 35 negative samples) was used for model training. Click here for file
