# Supplementary material for: In silico method for systematic analysis of feature importance in microRNA-mRNA interactions
Source: BMC Bioinformatics. 2009 Dec 16;10:427. doi: 10.1186/1471-2105-10-427 (PMC3087347; doi:10.1186/1471-2105-10-427)
Supplement: Additional file 1 — Table S1. Comparison of OOB and CV error estimate. [file 1471-2105-10-427-S1.DOC]

## Table S1 Comparison of OOB and CV error estimate.

| Feature set | Neg_1 | | Neg_2 | | Formula |
| --- | --- | --- | --- | --- | --- |
|  | Se | Sp | Se | Sp |  |
| SEQ | 0.884/0.873 | 0.835/0.828 | 0.835/0.821 | 0.890/0.885 | Se=TP/(TP+FN)  Sp=TN/(TN+FP) |
| STUR | 0.871/0.852 | 0.826/0.826 | 0.813/0.807 | 0.807/0.808 |  |
| POSI | 0.946/0.947 | 0.930/0.916 | 0.921/0.917 | 0.957/0.949 |  |
| Total | 0.967/0.971 | 0.934/0.918 | 0.888/0.870 | 0.934/0.922 |  |

The out-of-bag (OOB) prediction error is a built-in measurement of the performance [33]. Hence, cross-validation (CV) was not necessary. However, our measures are based on the predictors' performance in the training set. For the purpose of knowing whether the predictors are over-fitted to the training set, 10-fold cross-validation estimates the predictors' performance. Our results strongly shown the OOB error was very similar to the classification error of CV.

The left numbers of oblique line are OOB prediction accuracy, the right numbers of oblique line are CV prediction accuracy. TP is the number of true positive; TN is the number of true negative; FP is the number of false positive; FN is the number of false negative.
