# Supplementary material for: In silico method for systematic analysis of feature importance in microRNA-mRNA interactions
Source: BMC Bioinformatics. 2009 Dec 16;10:427. doi: 10.1186/1471-2105-10-427 (PMC3087347; doi:10.1186/1471-2105-10-427)
Supplement: Additional file 4 — Table S2. Prediction results of RF using experimental data. A very imbalanced experimental dataset (259 positive and 35 negative samples) was used for model training. [file 1471-2105-10-427-S4.DOC]

## Table S2 The prediction results of RF using experimental data.

| Feature set | Parameters | Sensitivity | Specificity | Accuracy |
| --- | --- | --- | --- | --- |
| SEQ | ntree=1000 mtry=9 | 0.992 | 0.686 | 95.67% |
| STUR | ntree=1000 mtry=9 | 0.966 | 0.686 | 93.29% |
| POSI | ntree=1000 mtry=5 | **0.974** | **0.800** | 95.33% |

A very imbalanced experimental dataset (259 positive and 35 negative) was used to train RF model. It is clear that the current set of 35 negative samples is not enough to represent the negative class, especially based on sequence and structural features. Therefore more negative data is required.

SEQ is the sequence feature set.

STUR is the structural feature set.

POSI is the positional feature set.
